# Implementation of a diabetic retinopathy referral network, Peru

**DOI:** 10.2471/BLT.18.212613

**Published:** 2018-08-27

**Authors:** Omar Salamanca, Amelia Geary, Nancy Suárez, Sara Benavent, Merly Gonzalez

**Affiliations:** a Orbis International, 520 8th Avenue, Floor 12, New York, NY 10018, United States of America.; b Instituto Regional de Oftalmología Javier Servat Univazo, Trujillo, La Libertad, Peru.

## Abstract

**Objective:**

To describe the implementation of a diabetic retinopathy referral network incorporating all levels of health care in La Libertad region, Peru.

**Method:**

The nongovernmental organization Orbis International and the Regional Institute of Ophthalmology established a network of primary, secondary and tertiary health-care facilities for diabetic retinopathy screening and treatment. The programme included the provision of three non-mydriatic retinal cameras for patient examination, the development of a flowchart to guide patient referrals, training of health personnel, investment in laser technology for treatment and the delivery of public awareness activities for blindness prevention and the need for timely screening.

**Findings:**

From 2014–2017, 11 849 patients with diabetes were screened within the diabetic retinopathy referral network. In primary-care centres, 6012 patients with diabetes mellitus were identified and 5632 patients were referred for diabetic retinopathy screening. A further 4036 patients directly attended two secondary-level hospitals and 2181 attended the tertiary-level hospital for screening. This represented a 138.1% increase in diabetic retinopathy screenings from a baseline of 4977 patients screened at the regional institute of ophthalmology over 2010–2013. A total of 2922 patients (24.7%) were found to have diabetic retinopathy and 923 (31.6%) were treated: 508 with laser photocoagulation, 345 with intravitreal bevacizumab and 70 with vitreoretinal surgery.

**Conclusion:**

Effective and timely treatment for diabetic retinopathy is possible when patient education, screening and care are fully integrated into the general health-care system across primary-, secondary- and tertiary-level facilities. This requires the integration of professionals at all levels and all relevant specialties.

## Résumé

**Objectif:**

Décrire la mise en place d'un réseau d'orientation pour la rétinopathie diabétique intégrant tous les niveaux de soins de santé dans la région de La Libertad, au Pérou.

**Méthodes:**

L'organisation non gouvernementale Orbis International et l'Institut régional d'ophtalmologie ont établi un réseau d'établissements de soins de santé primaires, secondaires et tertiaires pour le dépistage et le traitement de la rétinopathie diabétique. Le programme incluait la fourniture de trois caméras rétiniennes non mydriatiques pour examiner les patients, l'élaboration d'un organigramme pour guider l'orientation des patients, la formation de personnels de santé, un investissement dans la technologie laser pour soigner les patients et la mise en œuvre d'activités pour sensibiliser la population à la prévention de la cécité et à la nécessité d'un dépistage en temps opportun.

**Résultats:**

Entre 2014 et 2017, 11 849 patients diabétiques ont été examinés dans le réseau d'orientation pour la rétinopathie diabétique. Dans les centres de soins primaires, 6012 patients atteints de diabète sucré ont été identifiés et 5632 ont été orientés vers un service de dépistage de la rétinopathie diabétique. 4036 autres patients se sont directement rendus dans deux hôpitaux de niveau secondaire et 2181 se sont rendus dans un hôpital de niveau tertiaire pour un dépistage. Cela représentait une augmentation de 138,1% pour le dépistage de la rétinopathie diabétique par rapport à une valeur de référence de 4977 patients examinés au sein de l'Institut régional d'ophtalmologie entre 2010 et 2013. Il a été constaté qu'un total de 2922 patients (24,7%) étaient atteints de rétinopathie diabétique, parmi lesquels 923 (31,6%) ont été traités: 508 par photocoagulation au laser, 345 par bevacizumab en injection intravitréenne et 70 par chirurgie vitréo-rétinienne.

**Conclusion:**

Il est possible de traiter efficacement et en temps opportun la rétinopathie diabétique lorsque la sensibilisation des patients, le dépistage et la prise en charge sont pleinement intégrés au système général des soins de santé dans l'ensemble des établissements de niveau primaire, secondaire et tertiaire. Cela nécessite l'intégration de professionnels à tous les niveaux et pour toutes les spécialités pertinentes.****

## Resumen

**Objetivo:**

Describir la implementación de una red de referencia para la retinopatía diabética que incorpore todos los niveles de atención médica en la región de La Libertad, Perú.

**Métodos:**

La organización no gubernamental Orbis International y el Instituto Regional de Oftalmología establecieron una red de centros de atención primaria, secundaria y terciaria para la detección y el tratamiento de la retinopatía diabética. El programa incluía la instalación de tres cámaras retinianas no midriáticas para examinar a los pacientes, la elaboración de un diagrama de flujo para orientar las remisiones de pacientes, la formación del personal sanitario, la inversión en tecnología láser para el tratamiento y la realización de actividades de concienciación pública sobre la prevención de la ceguera y la necesidad de exámenes puntuales.

**Resultados:**

Entre 2014 y 2017, se examinaron 11 849 pacientes con diabetes en la red de referencia para la retinopatía diabética. En los centros de atención primaria se identificaron 6012 pacientes con diabetes mellitus y se derivó a 5632 pacientes para el examen de detección de la retinopatía diabética. Otros 4036 pacientes acudieron directamente a dos hospitales de nivel secundario y 2181 acudieron al hospital de nivel terciario para ser examinados. Esto representó un aumento del 138,1 % en las revisiones de retinopatía diabética a partir de una base de 4977 pacientes examinados en el instituto regional de oftalmología entre 2010 y 2013. Se concluyó que un total de 2922 pacientes (24,7 %) padecían retinopatía diabética y 923 (31,6 %) fueron tratados: 508 con fotocoagulación láser, 345 con bevacizumab intravítreo y 70 con cirugía vitreorretinal.

**Conclusión:**

El tratamiento eficaz y oportuno de la retinopatía diabética es posible cuando la educación, los exámenes de detección y la atención al paciente están plenamente integrados en el sistema general de atención sanitaria en los centros de nivel primario, secundario y terciario. Para ello se requiere la integración de profesionales de todos los niveles y todas las especialidades pertinentes.

## ملخص

الغرض وصف تنفيذ شبكة الإحالة لعلاج اعتلال الشبكية السكري، التي تتضمن جميع مستويات الرعاية الصحية في منطقة لا ليبرتاد في بيرو.

**الطريقة:**

قامت كل من المنظمة غير الحكومية Orbis International، والمعهد الإقليمي لطب العيون، بإنشاء شبكة من مرافق الرعاية الصحية الأولية والثانوية وفوق الثانوية لفحص اعتلال الشبكية السكري وعلاجه. ****وشمل البرنامج توفير ثلاثة كاميرات شبكية غير موسعة للحدقة لفحص المرضى، ووضع مخطط انسيابي لتوجيه إحالات المرضى، وتدريب فريق العمل الصحي، والاستثمار العلاج باستخدام تكنولوجيا الليزر، وتقديم أنشطة التوعية العامة للوقاية من العمى والحاجة للفحص في الوقت المناسب.

**النتائج:**

خلال الفترة من 2014 إلى 2017، تم فحص 11849 مريضاً مصاباً بداء السكري من خلال شبكة الإحالة لعلاج اعتلال الشبكية السكري. ****في مراكز الرعاية الأولية، تم اكتشاف إصابة 6012 مريضاً بالسكري، وتمت إحالة 5632 مريضا لفحص اعتلال الشبكية السكري. ****ودخل 4036 مريضاً آخرين مباشرة إلى مستشفيين من المستوى الثانوي، ودخل 2181 مريضاً مستشفى من المستوى فوق الثانوي، للخضوع للفحص. ****ومثل هذا زيادة قدرها 138.1٪ في فحوص اعتلال الشبكية السكري من خط الأساس بلغ 4977 مريضاً تم فحصهم في المعهد الإقليمي لطب العيون خلال الفترة من 2010 إلى 2013. تم اكتشاف أن إجمالي 2922 مريضاً (24.7٪) مصابين باعتلال الشبكية السكري، و923 مريضاً (31.6٪) تم علاجهم: ****عولج 508 مريضاً منهم بالتخثير الضوئي بالليزر، و345 مريضاً باستخدام بيفاسيزوماب داخل الجسم الزجاجي، و70 مريضاً بجراحة في الجسم الزجاجي بالشبكية.

**الاستنتاج:**

من الممكن علاج اعتلال الشبكية السكري بشكل فعال وفي الوقت المناسب، وذلك عند تحقيق التكامل بين تعليم المريض والفحص والرعاية بشكل كامل، في نظام الرعاية الصحية العام في المرافق الأولية والثانوية وفوق الثانوية. ****ويتطلب ذلك تحقيق التكامل بين المتخصصين على جميع المستويات، وفي جميع التخصصات ذات الصلة. ****

## 摘要

**目的:**

介绍秘鲁拉利伯塔德地区各级医疗保健的糖尿病性视网膜病变转诊网络的实施情况。

**方法:**

非政府组织奧比斯 (Orbis International) 和秘鲁眼科培训组织 The Regional Institute of Ophthalmology 建立了一个初级、二级和三级医疗保健设施网络，用于筛查和治疗糖尿病性视网膜病变。该项目包括：为患者检查提供三台非散瞳视网膜摄像机，制定指导患者转诊的流程图，培训保健人员，投资激光治疗技术，开展活动以提高公众预防盲症的意识和及时筛查的必要性。

**结果:**

自 2014 至 2017 年，有 11849 名糖尿病患者接受了糖尿病性视网膜病变转诊网络内的筛查。在初级保健中心，6012 名糖尿病患者中有 5632 名接受了糖尿病性视网膜病变的筛查。另有 4036 名患者直接于二级医院就诊，2181 名患者参加了三级医院的筛查。自 2010 至 2013 年，The Regional Institute of Ophthalmology 共筛查 4977 名患者，从该基线值可知筛查出的糖尿病性视网膜病变增加了 138.1%。共 2922 名患者（占 24.7%）被确诊为糖尿病性视网膜病变，其中有 923 名患者（占 31.6%）得到治疗：激光光凝术治疗 508 例，玻璃体内注射贝伐单抗 345 例，玻璃体视网膜手术 70 例。

**结论:**

将患者的教育水平、筛查和保健与一般医疗保健系统的初级、二级和三级设施充分结合，可以有效、及时地治疗糖尿病性视网膜病变。这需要各级专业人员和所有相关领域专家的精诚合作、通力配合。

## Резюме

**Цель:**

Описать реализацию реферальной сети медицинских учреждений, специализирующихся на диагностике и лечении диабетической ретинопатии, включающей все уровни системы здравоохранения в регионе Ла-Либертад, Перу.

**Метод:**

Неправительственная организация Orbis International и Региональный институт офтальмологии создали сеть медицинских учреждений первичного, вторичного и третичного уровня оказания медицинской помощи для проведения скрининга и лечения диабетической ретинопатии. Программа включала предоставление трех аппаратов для фотографирования сетчатки глаза, не требующих применения мидриатических препаратов, для обследования пациентов, разработку блок-схемы направления пациентов на лечение, обучение медицинского персонала, инвестиции в лазерную технологию для лечения и проведение мероприятий по информированию общественности о профилактике слепоты и необходимости своевременного скрининга.

**Результаты:**

С 2014 по 2017 год 11 849 пациентов с сахарным диабетом прошли обследование в реферальной сети медицинских учреждений, специализирующихся на диагностике и лечении диабетической ретинопатии. В центрах первичной медико-санитарной помощи было выявлено 6012 пациентов с сахарным диабетом и 5632 пациента были направлены на скрининг диабетической ретинопатии. Еще 4036 пациентов прошли скрининг в двух больницах вторичного уровня, а 2181 пациент — в больнице третичного уровня. Таким образом, было продемонстрировано увеличение частоты проведения скрининга диабетической ретинопатии на 138,1% по сравнению с исходными 4977 пациентами, которые прошли проверку в областном институте офтальмологии в период с 2010 по 2013 год. В общей сложности у 2922 пациентов (24,7%) была выявлена диабетическая ретинопатия и 923 пациента (31,6%) прошли лечение: 508 — с применением лазерной фотокоагуляции, 345 — с применением интравитреального введения бевацизумаба, 70 — с применением витреоретинальной хирургии.

**Вывод:**

Эффективное и своевременное лечение диабетической ретинопатии возможно в условиях, когда просвещение, скрининг и лечение пациентов полностью интегрированы в общую систему здравоохранения в учреждениях первичного, вторичного и третичного уровня оказания медицинской помощи. Это требует взаимодействия профессионалов всех соответствующих специальностей на всех уровнях.

## Introduction

The prevalence of diabetes mellitus is growing, particularly in low- and middle-income countries, and complications due to the disease are becoming major health issues requiring effective health interventions for prevention and treatment. An important vascular complication of diabetes is diabetic retinopathy, which affects the vision of 2.6 million people in the world,^1^ and is responsible for 2.6% of global blindness (0.84 million of 32.4 million people).^2^ The rise of diabetic retinopathy requires programmes for early detection, accurate diagnosis and timely treatment to reduce the impact of associated vision loss. The main objectives of a diabetes programme are to set norms and standards, promote surveillance, encourage prevention, raise awareness and strengthen prevention and control.^3^ The establishment of comprehensive programmes for the detection and management of diabetic retinopathy have been effective in decreasing the incidence of this complication.^4^

In Latin America, an estimated 29.6 million people were living with diabetes in 2015, or 9.4% of the adult population of 315 000 000.^5^ Screening can identify retinopathy in as much as 30% of patients with diabetes, and 5% of patients with diabetes are likely to need laser photocoagulation treatment to reduce the risk of blindness.^6^ Diabetic retinopathy is the third highest cause of functional vision loss in people older than 50 years, corresponding to 173 out of 910 (19%) of patients.^7^

The International Diabetes Federation reported a diabetes prevalence of 6.8% (1 248 822/18 365 030) for Peru in 2012. The prevalence of diabetic retinopathy in Peru has been estimated at between 23.1% (282/1222) and 30.0% (254/849) of patients with diabetes, who are twice as likely to be blind compared with those who do not have diabetes.^8^ Although access to health services in Peru has improved from 1990 to 2015,^9^ there are still challenges to accessing health services for many people, especially in the most remote rural areas and in low-income populations.^10^ Peru’s concerted national health plan 2007–2020 provided technical advice at the national level and identified diabetes mellitus as a priority for the health agenda. Also, visual impairment represents the second most frequent cause of disability in Peru.^11^

Trujillo is the capital of the La Libertad region of Peru and the third most populous city in Peru, with 799 550 inhabitants in 2015. The regional institute of ophthalmology (*Instituto Regional de Oftalmología Javier Servat Univazo*) provides comprehensive ophthalmological services for poorer patients. Since 2003, the institute has worked closely with the nongovernmental organization Orbis International, which has positioned the institute as the leading eye hospital and referral centre in northern Peru. In 2014, Orbis International and the institute partnered on a 4-year (2014–2017) project for early detection and referral for diabetic retinopathy with continuous follow-up and data collection. The project involved creation of a diabetic retinopathy referral and treatment network across primary, secondary and tertiary health-care facilities in northern Peru. This paper aims to describe the implementation of the network. We outline the project components and their implementation and an assessment of changes in early detection and referral of diabetic retinopathy four years after implementation.

## Methods

### Project implementation

The project objectives included: (i) to provide access to ocular care to the low-income population with diabetes and to offer early assessment and referral for timely treatment; (ii) to establish a health-care network for the referral of patients with diabetic retinopathy for screening, evaluation and ocular diagnosis, and treatment if required; and (iii) to strengthen the technical capacity of medical teams, providing adequate training to improve the efficiency and quality of diabetic retinopathy services.

#### Referral network

La Libertad region is divided into 12 primary-care networks, representing the 12 provinces. We included three of the 12 health networks in this project: Trujillo, Ascope and Virú. Within the three networks we included 10 primary health-care centres in Trujillo, one in Ascope and one in Virú (12 facilities in total). These facilities serve 94 satellite health posts. The catchment population of the included facilities is 1 530 652, which corresponds to 62.8% of the total regional population of 2 438 718. 

Before the project started, nurses at the primary health-care centres maintained a census of the names of patients living with diabetes, according to health ministry’s regulations on the care of noncommunicable diseases;^12^ this list is updated and reported every 3 months. Staff members at the institute collated the list across the 12 primary health-care centres quarterly to establish the population of patients with diabetes covered by the proposed diabetic retinopathy health network over the 4 years.

At the start of the project, the institute and Orbis International advocated directly with the regional health directorate to agree to the establishment of the diabetic retinopathy screening network. Care guidelines followed international recommendations^12^ and national regulations^13^ on health strategies for noncommunicable diseases. The network comprised the 12 primary health-care centres; two secondary-level hospitals (Docente de Trujillo regional hospital and Belén de Trujillo hospital); and one tertiary-level specialty hospital (regional institute of ophthalmology).

**Fig. 1:**
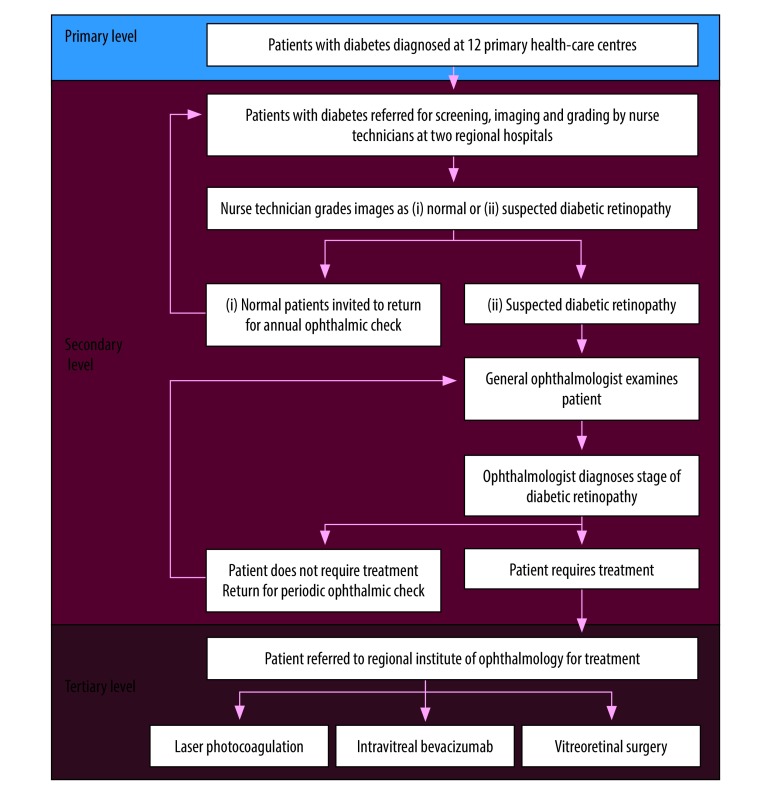
Flowchart of patients in the diabetic retinopathy referral network in northern Peru

With the support of Orbis International, equipment was procured for the start of the project in 2014. Three non-mydriatic retinal cameras (Topcon TRC-NW8, Topcon Medical Systems, Inc., Oakland, United States of America) were acquired for eye fundus examination and installed in the three referral hospitals. A laser for panretinal photocoagulation for diabetic retinopathy treatment (Nidek GYC-1000, Nidek Inc. Fremont, USA) was installed at the regional institute of ophthalmology. A flowchart was established by Orbis International and the institute to guide the referral of all identified patients with diabetes for an ophthalmological examination and treatment if required. Patients were diagnosed with diabetes at primary-care centres, referred to secondary level for diabetic retinopathy screening, and only patients requiring treatment or further diagnostic examinations were referred to tertiary-level care (Fig. 1).

#### Training

Staff members at the institute provided training for primary health-care teams, including general practitioners, nurses and technical staff on diabetes mellitus, diabetic retinopathy and the importance of timely referral. The institute’s head of the retina department and the director of training delivered training through interactive workshops, which lasted a total of 2 hours and were held once per year over the 4-year project duration. Training topics included epidemiology, diagnosis, treatment and prevention of diabetic retinopathy, health-team activities and use of the established referral flowcharts for patients with diabetic retinopathy. We developed a manual on diabetic retinopathy screening and distributed it at training events, to serve as a reference guide for primary health-care staff. Additionally, we trained nurses assigned to register patients with diabetes to refer patients to secondary-level hospitals. The nurse coordinator of the project monitored the impact of this strategy through personalized interviews with the participants.

Training at the secondary level focused on ophthalmic nurses and technicians, who were educated on image capturing with the non-mydriatic cameras and simple grading, triaging the results into normal and not normal. Not normal was confirmed by secondary-level ophthalmologists, who we trained on grading images and diagnosis of diabetic retinopathy. Patients requiring treatment were then referred to the tertiary level. To monitor the quality of the image capturing and simple grading by the trained technical personnel, a randomized subset of 100 photos was evaluated by two retina specialists who calculated the kappa index to measure interobserver concordance. Based on the findings, we carried out retraining, focusing on the weak points. Participants at these two levels did not receive extra income for participating in the project, but have benefited from educational activities at the local and regional level, aimed at improving the quality of life of their patients.

Training at the tertiary level focused on retina specialists, providing hands-on training in diabetic retinopathy treatment through laser therapy, intravitreal bevacizumab and vitreoretinal surgery. Volunteer expert faculty from Orbis International delivered five high-level training programmes between 2014 and 2016, consisting of the transfer of clinical and surgical skills. This programme included the use of advanced diagnostic technology and the management of complicated diabetic retinopathy cases. Constant monitoring and capacity-building for health-care staff at every level of care aimed to ensure effective knowledge transfer and that the skills developed were sustained and institutionalized.

#### Public awareness

The institute engaged in ongoing public awareness activities on diabetes mellitus, diabetic retinopathy and other ocular conditions, via social media, outreach campaigns, radio, television and distribution of educational materials. Awareness activities aimed to inform and educate the general public on diabetes, related avoidable blindness and visual impairment; the existence of the diabetic retinopathy network; and the importance of obtaining an ocular examination.

### Data collection

Seguro Integral de Salud is a free public health insurance provided by the Peruvian government for patients with low income, and which covers most costs associated with eye health. Orbis International used possession of the insurance as a proxy measure for low socioeconomic status among patients.

We consolidated the census of patients with diabetes and delivered it to the coordinating centre in the institute, where the information of all the health centres was reviewed and updated. At the secondary- and tertiary-level hospitals we collected and collated demographic data on patients screened, including sex, age, new or follow-up patient and having public health insurance or not. At the tertiary hospital, we collected the same demographic information for patients treated, in addition to the type of treatment. Facility registries and charts were the source of patient data. We made a clinical audit of the activities performed on patients with diabetes by making a random selection of the 81 clinical charts (8.7% of the 923 patients treated) and evaluating the institutions’ adherence to the protocols. From this information, we obtained demographic data on the patients and institutions, and made a descriptive analysis. We estimated the number of people reached by public awareness activities from the population that subscribed to the different social media outlets used for dissemination of public awareness campaign (e.g. number of followers on the institute’s Facebook page). We conducted a survey of patient satisfaction, but the data were insufficient to report here.

## Results

**Fig. 2:**
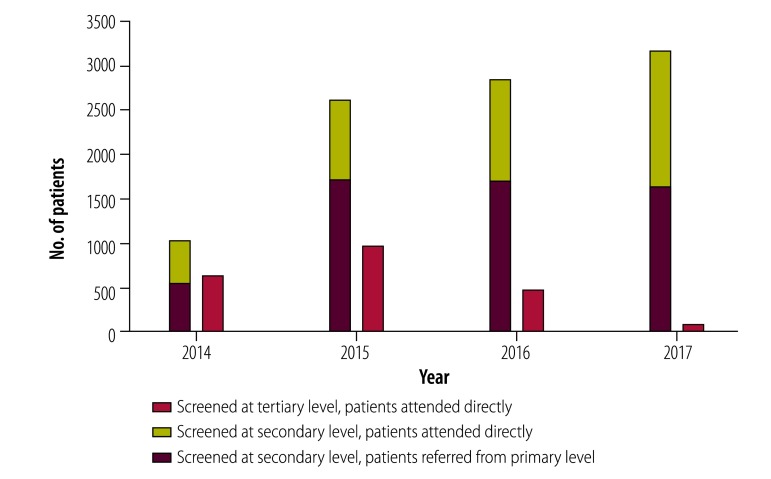
Patients with diabetes screened for diabetic retinopathy at different levels of health facility in northern Peru, 2014–2017

Notes: From 2014 and 2017, a total of 11 849 patients were screened at secondary and tertiary hospitals using non-mydriatic cameras. The diabetic retinopathy screening network comprised 12 primary health-care centres, serving a catchment population of 1 530 652, two secondary-level hospitals and one tertiary-level specialty hospital (regional institute of ophthalmology).

A total of 426 medical professionals were trained, including 323 general physicians, 27 ophthalmologists, 29 resident physicians, 30 nurses and 17 technicians. In the first 4 years of the project, a total of 11 849 patients with diabetes were screened with non-mydriatic cameras via the diabetic retinopathy network (Fig. 2). This compares with 4977 patients screened at the institute over 2010–2013, an increase of 138.1%. Of the patients screened, 9486 (80.1%) were screened for the first time and 2363 (21.9%) were follow-up patients. Of the 9066 patients that we collected demographic data on, 5629 (62.1%) were female and 3437 (37.9%) were male, with a mean age of 58.7 years. A total of 7900 (66.7%) corresponded to the target population: patients with diabetes attending public health-care facilities and of low socioeconomic status.

Primary-care facilities identified 6012 patients with diabetes (Table 1) and referred 5632 for retinopathy screening at secondary-level hospitals: 4204 (74.6%) attending for the first diabetic retinopathy screening and 1428 (25.4%) follow-up patients. A total of 4036 patients went directly to secondary-level hospitals and 2181 to the tertiary hospital. Screenings at secondary facilities increased from 1026 in 2014 to 3168 in 2017 and reduced at tertiary level from 638 in 2014 to 88 in 2017, effectively shifting the burden of screening and initial ocular evaluation from tertiary to secondary hospitals.

**Table 1:** Patients with diabetes screened and treated through the diabetic retinopathy network in northern Peru, by primary health-care centre, 2014–2017

Primary health-care centre	Projected population aged > 30 years^a^	Cumulative census of registered patients with diabetes, 2014–2017	No. of new patients screened for diabetic retinopathy at referral hospitals	No. of follow-up patients screened for diabetic retinopathy at referral hospitals	No. of patients treated at regional institute of ophthalmology
Alto Trujillo	4 002	217	141	38	12
Ascope	2 039	428	267	78	37
Cruz Vilca	3 458	499	283	81	16
El Esfuerzo	6 114	577	354	123	22
El Milagro	5 021	473	332	125	36
Jerusalén	11 667	787	685	273	94
La Noria	33 649	939	818	271	74
Laredo	3 351	393	196	97	23
Santa Isabel	9 902	693	487	151	53
Virú	5 427	341	154	26	19
Vista Alegre	6 193	491	369	113	40
Wichanzao	2 994	174	118	52	15
**Total**	**93 817**	**6 012**	**4 204**	**1 428**	**441**

**^a^** Data from the Seguro Integral de Salud public health insurance records.

**Fig. 3:**
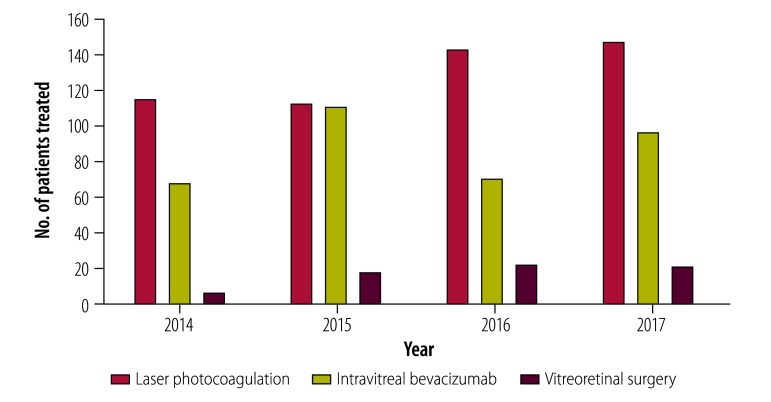
Patients with diabetes referred for treatment of diabetic retinopathy at the tertiary-level regional institute of ophthalmology in northern Peru, 2014–2017

Notes: From 2014 and 2017, a total of 11 849 patients were screened for diabetic retinopathy at 12 primary health-care centres, serving a catchment population of 1 530 652, two secondary-level hospitals and the regional institute of ophthalmology.

A total of 2922 patients (24.7%) were found to have diabetic retinopathy. The institute treated 923 patients referred through the network (which represented 30.2% of all 3057 diabetic retinopathy patients treated at the institute; Fig. 3): 508 for laser treatment, 345 for intravitreal bevacizumab and 70 for vitreoretinal surgery (we only counted patients who received more than one treatment once).

Of 100 images assessed, 85 were gradable, 12 had high opacity and 3 were not gradable. The evaluation of the accuracy of image grading showed the level of agreement between trained nurses and ophthalmologists was substantial to moderate, with a kappa value of 0.61 (data are available from the corresponding author).

Public awareness activities reached an estimated 119 153 people and could have been a factor in increased screening at primary and secondary level facilities; however, the impact of these interventions is unknown.

The total investment for the 4-year project was 2 344 559 United States dollars (US$), partially covered by Orbis International (US$ 1 507 551; 64.3%) and partially covered by the institute (US$ 837 007; 35.7%), reflecting the strong partnership component at all levels. These costs were distributed as follows: equipment US$ 721 000, medical supplies US$ 977 668, training US$ 400 885, staffing and professional fees US$ 167 550 and other US$ 77 456.

## Discussion

A comprehensive treatment network for diabetic retinopathy requires ocular care to be integrated into every level of health care. This integration requires that health professionals at primary, secondary and tertiary health-care centres have the appropriate knowledge, skills and infrastructure to identify, diagnose, refer and treat diabetic retinopathy. Our strategies included widespread multidisciplinary training and skills development; the procurement of adequate and cost-efficient technology; advocating with the government; and adequate public awareness plans. The diabetic retinopathy referral network increased the screening and treatment of patients with diabetes without further stretching the resources at specialized facilities.

Compared with other studies in Latin America using non-mydriatic cameras, such as in Costa Rica,^14^ we screened a larger number of patients with diabetes over a longer period of time. However, the challenge remains to ensure the sustainability of the referral network over time. It has been suggested that programmes to reduce diabetic retinopathy incidence must be planned for 10 years, ideally with diabetes educators,^6^ implying additional phases to the project might be required to sustain the results. We made efforts to ensure sustainability, however. The institute benefitted from continued training of their medical and allied health staff throughout the course of the project. This resulted in the institute acquiring the capacity to uphold the quantity and quality of eye health-care services achieved beyond the end of the project. The institute also increased its financial revenues by successfully tapping into the public health insurance system to support the costs of providing services to patients from low socioeconomic backgrounds. Additionally, the donated equipment stayed at the institute and the network partners. The estimated lifespan for fundus cameras is 8 years and for laser equipment is at least a decade.^15^ We also trained biomedical engineers at the partner institutions to ensure maintenance of this equipment.

An important feature of this project was the training of medical and allied health personnel throughout the referral network, which many believe is critical for this type of activity to be successful. Efforts are needed to expand the network beyond ophthalmology, to include general medicine and endocrinology, and health professionals of all disciplines, including nurses, technicians, counsellors and doctors. Such expansion would allow for integration of screening within the basic care programmes of diabetes mellitus, streamlining patient care and examinations and leading to integrated management of patients with diabetes.^16^ However, the persistent turnover and relocation of staff at primary-care centres in La Libertad region, proved a constant challenge to training and capacity-building efforts.

Delivering diabetic retinopathy screening at the primary-care level could further increase the number of patients screened and reduce attrition. However, the challenges of a diabetic retinopathy detection programme at this level include the lack of both financial resources and qualified staff. With the use of non-mydriatic cameras, diabetic retinopathy screening programmes are showing success at using adequately trained non-ophthalmologists to capture and grade images.^16^^–^^18^ Additionally, the health technology industry continues to produce lower cost image-capturing devices, which may drive down the technological costs of screening.^19^ Combining telemedicine with the use of these cameras, and connecting health centres to an expert reading centre and personnel, can improve diabetic retinopathy screening, and has been adopted by some national screening programmes.^20^ A subcomponent of this, tele-education, can be useful to improve diagnostic performance in diabetic retinopathy. Particularly in middle-income countries, tele-education may represent a key strategic intervention to improve the quality and quantity of diabetic retinopathy care providers without an added burden on existing local human and educational resources.^21^ In addition, integrating diabetic retinopathy screening in general medicine and endocrinology clinics and into primary care has demonstrated the effectiveness of screening strategies using cameras.^22^^–^^25^

This study has several limitations, mainly due to gaps in data collection. It is evident that there has been an increase in the number of patients screened and therefore referred for treatment. However, there are not enough data to determine the effectiveness of the project in relation to the prevention of visual impairment and its global impact on blindness in northern Peru. Furthermore, while patient volume and demographics were collected at each level of care, data related to patients who did not complete the referral pathway were not collected. Such data would include why patients identified at primary-care level and diagnosed with diabetes did not receive an eye examination at secondary-level hospitals; and why patients identified at secondary level requiring treatment did not pursue treatment at the tertiary level. Towards the end of the project, staff at the regional institute of ophthalmology called 26 patients referred for treatment who did not attend their screening appointments and found that most patients prioritized treatment of other health issues perceived to be more serious. Finally, many characteristics of the underlying disease remain unknown, including duration, severity, treatment and if it is a first-time diagnosis: information that is important for diabetes mellitus in relation to visual prognosis. Going forward, these data would be required to assess both the effectiveness of the model and identify strategies to further increase coverage.

In summary, the collaboration between Orbis International and the regional institute of ophthalmology in La Libertad has successfully established an efficient diabetic retinopathy referral system in northern Peru. The project has increased the number of known persons living with diabetes who have received an eye examination at both secondary- and tertiary-level hospitals and provided timely and accurate detection and treatment of diabetic retinopathy. The project demonstrates that effective treatment for diabetic retinopathy is possible when education, screening and care are fully integrated into the general health-care system.
